# Late Adulthood, COVID-19-Related Stress Perceptions, Meaning in Life, and Forgiveness as Predictors of Mental Health During the COVID-19 Pandemic

**DOI:** 10.3389/fpsyg.2021.731017

**Published:** 2021-09-29

**Authors:** Loren Toussaint, Alyssa Cheadle, Jessie Dezutter, David R. Williams

**Affiliations:** ^1^Department of Psychology, Luther College, Decorah, IA, United States; ^2^Department of Psychology, Hope College, Holland, MI, United States; ^3^Meaning Research Late Life Lab, School Psychology & Development in Context, University of Leuven, Leuven, Belgium; ^4^Department of African and African American Studies, Harvard University, Cambridge, MA, United States; ^5^Department of Social and Behavioral Sciences, Harvard T.H. Chan School of Public Health, Boston, MA, United States

**Keywords:** old age, late adulthood, forgiveness, meaning in life, mental health, stress appraisals, COVID-19

## Abstract

The purpose of this study was to examine multiple direct and indirect pathways of the association between older age and mental health through COVID-19-related stress perceptions, meaning in life, and forgiveness of situations. Participants were 1,382 U.S. adults who were part of the Harvard Digital Lab for the Social Sciences panel who volunteered to complete a 12 min survey in the spring of 2020. The sample had an average age of 56 years, was slightly more male (55%) than female (44%) or other (2%), mostly White (88%), well-educated (70% bachelors degree or more), and middle-income ($60,000–$75,000 annually). Measures included: COVID-19-related stress perceptions (e.g., concerns about infection, job, lack of necessities), presence of and search for meaning in life, forgiveness of situations, psychological distress, hopelessness, and optimism. A latent mental health variable was created that was comprised of psychological distress, hopelessness, and optimism. All hypothesized direct effects were in evidence, and all but one indirect effect were observed. Specifically, older age was related to better mental health through higher presence of meaning and lower search for meaning. Older age was also related to better mental health through a serial indirect pathway from lower COVID-19-related stress perceptions to higher presence of and lower search for meaning and higher forgiveness of situations to mental health. The proposed model was largely supported and confirms existing theory and research on aging, positive psychological processes, and mental health. Findings also offer new insights on the unique potential role of forgiveness of situations and its theoretical relevance to offending situations such as the COVID-19 pandemic. The present study offers a beginning for theorists, researchers, and practitioners to consider the connections between aging and mental health and the intricate interconnections between stress appraisal and positive coping resources that may serve to support it.

## Introduction

The COVID-19 pandemic presents challenges in all walks of life for people around the world (American Psychological Association, 2020; Pandey et al., 2020; Varga et al., 2021). Often these challenges are either buffered or exacerbated by socio-demographic variables (e.g., net worth, social support, and job-security) (McKnight-Eily et al., 2021). One notable example is the importance of age in terms of one's risk for contracting the coronavirus (The Novel Coronavirus Pneumonia Emergency Response Epidemiology Team, 2020) and experiencing adverse outcomes including mortality (Nanda et al., 2021). However, while advanced age may create biological vulnerability to disease including COVID-19, generally older adults report good mental health (Westerhof and Keyes, 2010). In the specific context of the COVID-19 pandemic, community-dwelling, older adults may experience fewer COVID-19-related stressors and appraise them as less threatening (Park et al., 2020). Beyond the effects of less exposure to and less threatening appraisals of common pandemic-related stressors such as job loss or lack of necessities, there may also be other mechanisms that could protect older adults' mental health during the COVID-19 pandemic. A key focus of the present study is to highlight the importance of two key psychosocial mechanisms for protecting older adults' mental health—meaning and forgiveness. More specifically, we will examine presence of and search for meaning in life and forgiveness of situations, an all too often overlooked or dimension of forgiveness that could be apropos to the COVID-19 pandemic circumstances. We chose to examine these two factors because older adults show higher levels of presence of meaning and lower levels of search for meaning (Steger et al., 2009) and higher levels of multiple forms of forgiveness (Toussaint et al., 2001). This raises two key questions. First, could the appraisal of less stress related to the COVID-19 pandemic serve as a mechanism for increased presence of meaning, decreased search for meaning, and increased forgiveness of situations? Second, could the resulting increased presence of meaning, decreased search for meaning, and increased forgiveness of the COVID situation have benefits for mental health?

### Late Adulthood, Stress, and Mental Health

Lifespan development theorists argue that development continues well into late adulthood. Notable theorists such as Carstensen et al. (2003), Charles (2010), Erikson (1982), and Tornstam (2005) offer perspectives that suggest that in later adulthood, aging individuals confront key challenges and can resolve them in ways that lead to mental health and well-being. More specifically, Charles (2010) suggests in her Strength and Vulnerability Integration model that older adults are more likely to engage strategies that limit negative experiences. Erikson (1982) suggests that the integrity versus despair stage of development occurs in late life and consists of feelings of regret about missed opportunities, unachieved goals, or lack of productivity (i.e., despair) or a sense of meaning, coherence, and satisfaction with how life has turned out (i.e., integrity). Tornstam (2005) argues in his Gerotranscendence theory that as individuals age, they become less self-centered and more other-oriented and transcendent in their perspective. Carstensen et al. (2003) suggests in their Socioemotional Selectivity Theory that as individuals age they prioritize meaningful goals and selectively invest in relationships and people who provide positive emotional outcomes. Collectively, these theories support the hypothesis that older adults, in the course of resolving late-life issues (Erikson), transforming perspectives and goals (Tornstam), avoiding negative stimuli and stressors (Charles), and selectively investing in relationships (Carstensen), might build personal and social resources that allow one to experience less threatening stress appraisals, cope better with stress, and enjoy better mental health and well-being.

Although the evidence is not entirely clear-cut (Jorm, 2000), considerable research does support the assertion that older adults experience less stress (Aldwin, 1990), less depression and enjoy more positive emotions (Blazer and Hybels, 2005; Kessler et al., 2010a). This has been especially true during the COVID-19 pandemic. For instance, older adults experienced such things as less stress, depression, and anxiety and better emotional well-being and resilient coping in several studies including: (a) a sample of older adults from 26 countries (Kowal et al., 2020), (b) an international sample from 63 countries (Varma et al., 2021), (c) a U.S. sample (Carstensen et al., 2020), and (d) a U.S. and Canadian sample (Klaiber et al., 2020). Additional U.S. studies showed that older adults reported less exposure to COVID-19-related stressors specifically, and importantly, rated COVID-19 events as less stressful (i.e., less threatening stress appraisals) (Park et al., 2020), and reported less negative impact of COVID-19 on well-being (Knepple Carney et al., 2021). In summary, both theoretical and empirical work suggest that older adults who progress through stages of late life development that lead to a sense of integrity, altruism, other-orientedness, and strong social support can enjoy less stress, less concerning stress appraisals, and better mental health and well-being. These advantages of late life have particular relevance for the COVID-19 pandemic because they facilitate adjustment during a time of unbearable stress and mental health crises (American Psychological Association, 2020).

### Late Adulthood, Stress, Meaning, and Forgiveness

From a broad view, much of the collective work done by Carstensen et al. (2003), Charles (2010), Erikson (1982), and Tornstam (2005) could be viewed as coalescing around the idea that individuals in later adulthood who have cognitive and emotional skills that promote positive aging can do better in coping with life stress and thereby experience better mental health. Two important implied aspects of these theories that are central to the present work are meaning and forgiveness. Meaning making and forgiveness are coping strategies relevant to the integrity-despair life stage (Erikson, 1982), are in support of the transformation from inward to outward other-focused thought and altruism (Tornstam, 2005), and are key to selectively investing and *maintaining* emotionally support social networks (Carstensen et al., 2003). Forgiveness, including forgiveness of situations, has been shown to be higher in older adults (Toussaint et al., 2001; Kaleta and Mróz, 2018) and an important part of the late life process of integrity and despair resolution which can contribute to better mental health (Dezutter et al., 2016; Derdaele et al., 2019). Presence of meaning in life has also been shown to be higher and search for meaning in life has been shown to be lower in older adults as compared to younger adults and both are correlated with mental health and emotional well-being in the late life stage (Steger et al., 2009). Importantly, these age differences in forgiveness and meaning may come about as a result of the late-life developmental changes discussed by lifespan theorists (Erikson, 1982; Carstensen et al., 2003; Tornstam, 2005; Charles, 2010) which may also modulate stress appraisal through more ego-integration, altruism, and social support, and hence make forgiveness and meaning in later years even more likely. Indeed, both the stress-and-coping theory of forgiveness (Worthington and Scherer, 2004) and logotherapy (Frankl, 1985) suggest that it is the appraisal of a stressful event or set of circumstances that prompts an individual to consider the need to engage in forgiveness or make sense of a life event or social circumstance that has caused harm to oneself or others.

### Meaning, Forgiveness, and Mental Health

Central to our understanding of forgiveness and meaning is that both processes are healthy, adaptive forms of coping with life events and circumstances deemed stressful. Indeed, making sense of one's life has been shown to be connected with improved mental and physical health (Cohen et al., 2016; Thir and Batthyány, 2016; Musich et al., 2018), and this is equally true for individuals in late life as in younger individuals (Steger et al., 2009). Results of a meta-analysis show that meaning is also inversely related to post-traumatic stress disorder symptom severity (Schäfer et al., 2019), and an additional meta-analysis shows that meaning is connected to a broad range of improved mental health and well-being outcomes including depression, satisfaction with life, pain, cognitive function, health-related quality of life, and several other health outcomes in older adults (Koelen et al., 2017). Similar to research on meaning and health, a good deal of research on forgiveness and mental health has shown that forgiveness is related to better mental health outcomes and these relations are at least as strong if not stronger for older adults (Toussaint et al., 2001; Webb and Toussaint, 2020). Much of the research on forgiveness and health relies on the stress-and-coping theory of forgiveness (Worthington and Scherer, 2004) to explain associations between the stress of unforgiveness, forgiveness, and mental health. That is, unforgiveness is considered stressful and can have deleterious connections to mental health, whereas the reverse is true for forgiveness which has a beneficial connection to mental health. In line with theories of late-life development (Erikson, 1982; Carstensen et al., 2003; Tornstam, 2005), if older adults are more inclined toward altruism and selective investment in relationships which they are highly motivated to maintain, then forgiveness may be especially helpful for older adults. It is important to note, however, that most research on forgiveness and health is focused on self-forgiveness or forgiveness of others and rarely does forgiveness of broader situations get considered. Bad situations can be viewed as offending an individual's sense of justice, just-world beliefs, or meaning (Thompson et al., 2005). As such, situations can be forgiven in a fashion roughly akin to self-forgiveness or forgiveness of others wherein the primary transformation is from negative thoughts, feelings, and motivations regarding the situation to more positive thoughts, feelings, and motivations. Offending situations abound, and exemplars would include catastrophic events such as natural disaster or terminal or chronic illness (e.g., COVID-19). It is uncanny that forgiveness has been theoretically and empirically tied to human-made and natural disasters and acts of god (Worthington Jr et al., 2016; Toussaint et al., 2017; Fincham and May, 2021), but consideration of forgiveness of situations and its role in resilience and recovery has been virtually absent. This is a significant omission in the literature. Hence, it is important to empirically examine forgiveness of situations and understand its potentially unique role in aging, stress, and mental health.

### A Comprehensive Model

Based on the review of the literature above, the model tested in this study examines two main research questions. First, we examine the association of older age and its relation to COVID-19-related stress perceptions, presence of and search for meaning, and forgiveness of situations. Further, we examine the extent to which age differences in presence of and search for meaning and forgiveness of situations might be indirectly conveyed through reduced COVID-19-related stress perceptions. Second, we examine indirect associations of age with mental health through presence of and search for meaning and forgiveness of situations, and further examine serial indirect associations from age to COVID-19-related stress perceptions to presence of and search for meaning and forgiveness of situations to mental health.

### Present Study

**Figure 1:**
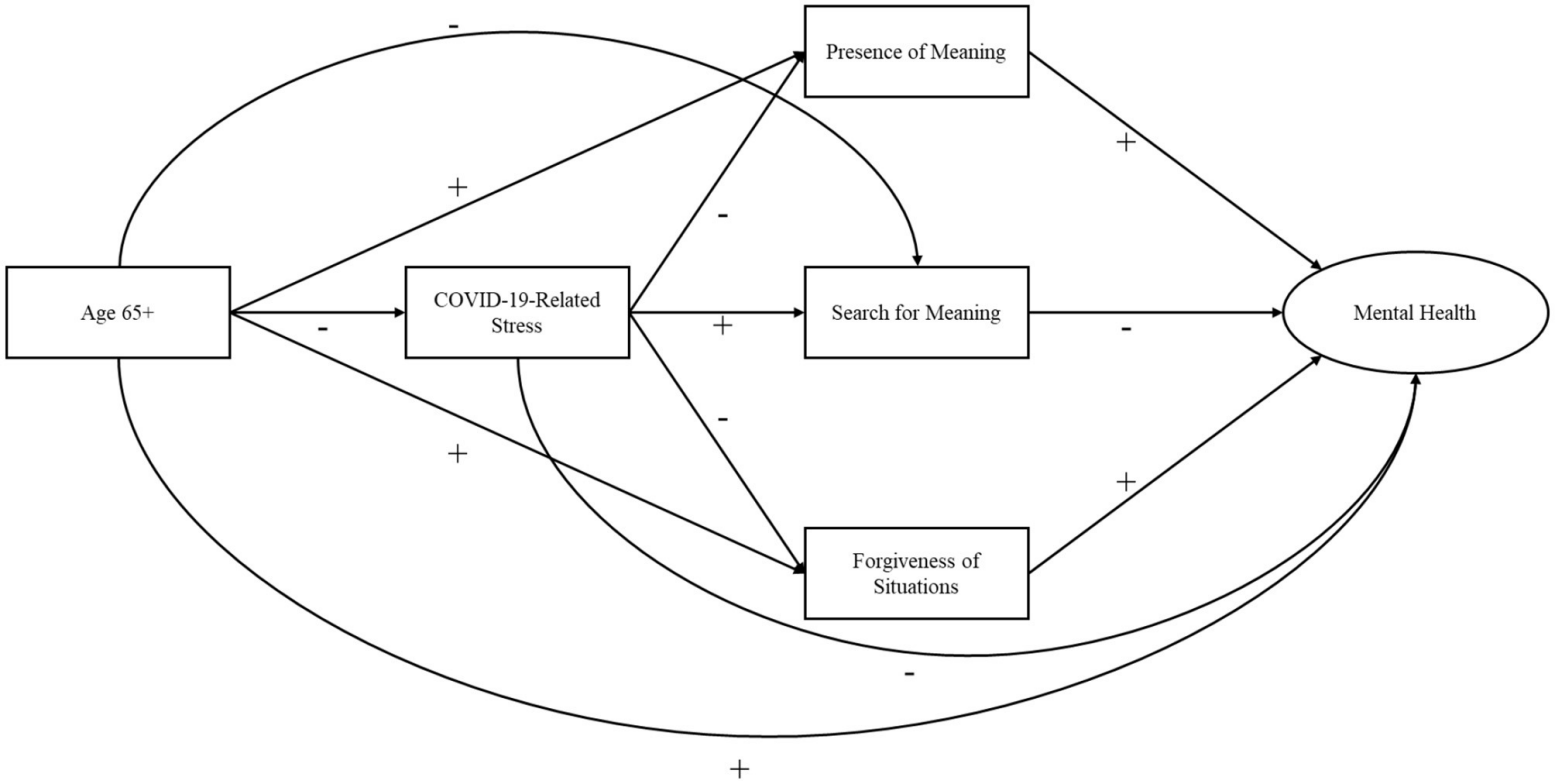
Hypothesized structural model. + and – indicate predicted positive and negative associations.

The present study examines a model of age, COVID-19-related stress perceptions, presence of and search for meaning, forgiveness of situations, and mental health in a national sample of U.S. residents who completed an online survey between April 17, 2020 and July 17, 2020. We hypothesize that individuals age 65 and over will have lower COVID-19-related stress perceptions and that these will allow greater presence of meaning and forgiveness of the situation as well as less search for meaning. Presence of meaning along with forgiveness of situations will be positively and search for meaning will be negatively related to mental health. As such we hypothesize all possible direct (see Figure 1) and 10 indirect (both single-mediator and serial mediator, see below) associations will be present in the model. The hypothesized indirect associations are as follows:

↑Age 65+ → ↓COVID-19 stress perceptions → ↑Mental health
↑Age 65+ → ↓COVID-19 stress perceptions → ↑Presence of meaning
↑Age 65+ → ↓COVID-19 stress perceptions → ↓Search for meaning
↑Age 65+ → ↓COVID-19 stress perceptions → ↑Forgiveness of situations
↑Age 65+ → ↑Presence of meaning → ↑Mental health
↑Age 65+ → ↓Search for meaning → ↑Mental health
↑Age 65+ → ↑Forgiveness of situations → ↑Mental health
↑Age 65+ → ↓COVID-19 stress perceptions → ↑Presence of meaning → ↑Mental health
↑Age 65+ → ↓COVID-19 stress perceptions → ↓Search for meaning → ↑Mental health
↑Age 65+ → ↓COVID-19 Stress Perceptions → ↑Forgiveness of Situations → ↑Mental Health

## Method

### Participants

Participants in this study were individuals who were part of the Harvard Digital Lab for the Social Sciences (DLABSS). This is an international panel of 16,000 volunteer respondents who participate in social science research. For the present study, a U.S. national sample of 1,382 respondents completed, on average, a 12 min survey between April 17, 2020 and July 17, 2020 that included multiple measures of health, psychosocial, religious and spiritual, and COVID-19-related stress perceptions and anxiety. Table 1 contains descriptive statistics for all socio-demographic variables. Participants had a median age of 56 years. In terms of sex, 41% were female, 55% were male, and 2% other. The predominant race was “White” (88%) while each of the other racial categories represented 5% or less of the sample. The sample was well-educated with ~70% of participants having an undergraduate or graduate degree. The median annual household income category (chosen from 25 categories) was $60,000–$75,000. Institutional review board approval was obtained for this study (IRB20-0599), and all participants were provided with informed consent.

**Table 1:** Socio-demographic characteristics.

**Variable**	***Mdn (N)***	**Range (%)**
Age (Missing—*N* = 23, 1.7%)	56.0	19–120
Sex		
Female	572	41.4
Male	764	55.3
Other/prefer not to answer	23	1.7
Race		
Asian	24	1.7
Black	27	2.0
Hispanic/Latino	28	2.0
Native American	7	0.5
Native Hawaiian or Pacific Islander	2	0.1
White	1,210	87.6
Other	67	4.8
No response	17	1.2
Education		
Elementary school	1	0.1
Some high school	5	0.4
High school graduate	224	16.2
Associate degree	178	12.9
Bachelor degree	474	34.3
Master degree	352	25.5
Doctoral degree	106	7.6
No response	42	3.0
Income (Missing—*N* = 149, 10.8%)	$60k–$75k	< = 3k−150k+

### Measures

#### Age

Respondents provided their birth year. Birth year was subtracted from 2020 to obtain an individual's age. Although age was collected and computed in this continuous form, for the purposes of this study and special issue, we chose to focus on the differences between individuals 65 years of age and older compared to adults who were younger than 65.

#### COVID-19-Related Stress Perceptions

COVID-19-related stress perceptions were assessed by asking respondents to indicate their levels of concern regarding four issues facing them in the pandemic. The issues included concerns about: oneself or one's family members being infected with the coronavirus, one's job, business, or personal finances, and running out of necessities like food, toilet paper, or medications. Each item was rated on a 1 (*not at all*) to 5 (*extremely*) response scale. Internal consistency for this scale in the present study was 0.67.

#### Meaning in Life

Meaning in life was assessed using four items from the Meaning in Life Questionnaire-Short Form (Steger and Samman, 2012). The original scale measures presence of and search for meaning in life with six items. Because of time constraints of the survey (12 mins or less required for adequate participation), we sought to shorten this assessment by 33% (two items). As all six items had very high loadings on their respective presence and meaning factors, we chose two items from each factor with the clearest face-validity. An example presence of meaning item is, “My life has a clear sense of purpose,” and an example search for meaning item is, “I am searching for meaning in my life.” The scale has excellent factorial and construct validity with measures of well-being. Each item was rated on a 1 (*absolutely untrue*) to 5 (*absolutely true*) response scale. Internal consistency of the presence and search subscales in the present study was 0.87 and 0.75, respectively.

#### Forgiveness of Situations

Forgiveness of situations was assessed using a shortened form of the scale developed by Thompson et al. (2005). Forgiveness of situations is conceived of by Thompson et al. (2005) as being similar to forgiveness of oneself and others because situations can create affronts to one's sense of justice or fairness and hence can be the source of offense. Victims of offensive situations might be those affected by natural or human-made disasters or may be patients diagnosed with terminal or chronic diseases, or may simply be an individual caught in adverse or challenging circumstances for which an easy target of blame cannot be identified. The forgiveness of situations scale was shortened from six to two items because of time constraints of the overall survey. Based on the results of factor analyses of four independent datasets totaling 4,050 respondents, we chose the consistently highest loading two items to include as the forgiveness of situations scale in this study. These items were: “With time I can be understanding of bad circumstances in my life,” and “I eventually make peace with bad situations in my life.” Each item was rated using the original response options given by Thompson et al. (2005) of 1 (*almost always false of me*) to 7 (*almost always true of me*). Internal consistency for this scale in the present study was 82.

#### Mental Health

Three aspects of mental health were assessed with the intent to create a latent variable that taps cognitive and emotional aspects of positive mental health and well-being. Psychological distress was measured using the Kessler-6 (Kessler et al., 2010a,b). This scale contains six items assessing key symptoms of non-specific psychological distress. Examples include feeling “nervous,” “hopeless,” and “worthless.” Each item was responded to with response options of 0 (*none of the time*) to 3 (*most of the time*). The Kessler-6 is one of the most commonly used measures of psychological distress and has excellent psychometric properties (National Comorbidity Survey, 2005). Internal consistency for this scale in the present study was 0.89.

Hopelessness was measured as the second component of mental health. We used a brief two-item index of hopelessness (Everson et al., 1996, 1997). The items are: “The future seems to me to be hopeless, and I can't believe that things are changing for the better,” and “I feel it is impossible for me to reach the goals that I would like to strive for.” Each item was responded to with 1 (*strongly disagree*) to 5 (*strongly agree*) response options. The scale has good construct validity and has been used in several population-based studies (Fraser et al., 2014). Internal consistency for this scale in the present study was 0.77.

Optimism was measured as the third component of mental health. We used the three-item optimism subscale of the Life Orientation Test developed by Scheier et al. (1994). An example item is, “In uncertain times, I usually expect the best.” Each item was responded to with 1 (*I disagree a lot*) to 4 (*I agree a lot*) response options. This scale is one of the most widely used measures of optimism and possesses excellent reliability and validity (Scheier et al., 1994). Internal consistency for this scale in the present study was 0.85.

#### Socio-Demographics

Socio-demographic variables (other than age) included sex, race, education, and income and were included as covariates in the model.

### Analyses

Descriptive statistics and bivariate analyses were conducted. The hypothesized model was tested using the lavaan package in R. The lavaan package offers the ability to test the proposed serial mediation model and examine direct, indirect, and serial indirect effects. Indirect effects were tested using bias-corrected bootstrapped confidence intervals (*N* = 1,000) as recommended by Hayes (2013). Model fit was determined using χ^2^, confirmatory fit index (CFI), root mean-square error of approximation (RMSEA), and standardized root mean residual (SRMR) and recommended by Kline (2015). Evidence of an acceptable-fitting model was taken by CFI ≥ 0.90, RMSEA ≤ 0.08, SRMR ≤ 0.08, and evidence of a good-fitting model was taken by CFI ≥ 0.95, RMSEA ≤ 0.05, SRMR ≤ 0.05 (Schermelleh-Engel et al., 2003; Kline, 2015). Power to reject an ill-fitting model (parameters: RMSEA = 0.08, *p* = 0.05, *N* = 1,382) was 0.99. Model estimation was performed using maximum likelihood with full information maximum likelihood used to account for missing data. The Bollen-Stine χ^2^ statistic (*p* < 0.0001) indicated a significant departure from multivariate normality so robust, bootstrap-adjusted fit indices are reported. All reported parameter estimates controlled for biological sex, race, education, and income. Statistical significance was set at *p* < 0.05.

## Results

Table 2 contains means, standard deviations, and bivariate correlations for main study variables. Participants aged 65 and older showed less COVID-19-related stress perceptions, search for meaning, psychological distress, and hopelessness (*r*s = −0.12 to −0.25), and more presence of meaning, forgiveness of situations, and optimism (*r*s = 0.08–0.26). More COVID-19-related stress perceptions was associated with less presence of meaning, forgiveness of situations, and optimism (*r*s = −0.12 to −0.37), and more search for meaning, psychological distress, and hopelessness (*r*s 0.18–0.53). More presence of meaning was associated with less search for meaning, psychological distress, and hopelessness (*r*s = −0.12 to −0.41), and more forgiveness of situations and optimism (*r*s = 0.32–0.46). Search for meaning was associated with more psychological distress and hopelessness (*r*s = 0.18–0.28) and less optimism (*r* = −0.14) but was unrelated to forgiveness of situations (*r* = −0.03). Forgiveness of situations was related to less psychological distress and hopelessness (*r*s −0.23 to −0.26) and more optimism (*r* = 0.38). Psychological distress was related to more hopelessness (*r* = 0.66) and less optimism (*r* = −0.54) and more hopelessness was related to less optimism (*r* = −0.58).

**Table 2:** Descriptive statistics and bivariate correlations for main study variables.

	**M**	**SD**	**1**.	**2**.	**3**.	**4**.	**5**.	**6**.	**7**.	**8**.
1. Age 65+	0.34	0.48	–							
2. COVID-19-related stress perceptions	2.61	0.82	−0.12***	–						
3. Presence of meaning	9.95	3.05	0.12***	−0.17***	–					
4. Search for meaning	8.28	3.25	−0.13***	0.18***	−0.12***	–				
5. Forgiveness of situations	10.74	2.55	0.08**	−0.12***	0.32***	−0.03	–			
6. Psychological distress	4.97	4.48	−0.25***	0.53***	−0.35***	0.28***	−0.23***	–		
7. Hopelessness	2.65	2.39	−0.19***	0.42***	−0.41***	0.18***	−0.26***	0.66***	–	
8. Optimism	8.57	2.41	0.26***	−0.37***	0.46***	−0.14***	0.38***	−0.54***	−0.58***	–

** *p < 0.01*,

*** *p < 0.001*.

**Figure 2:**
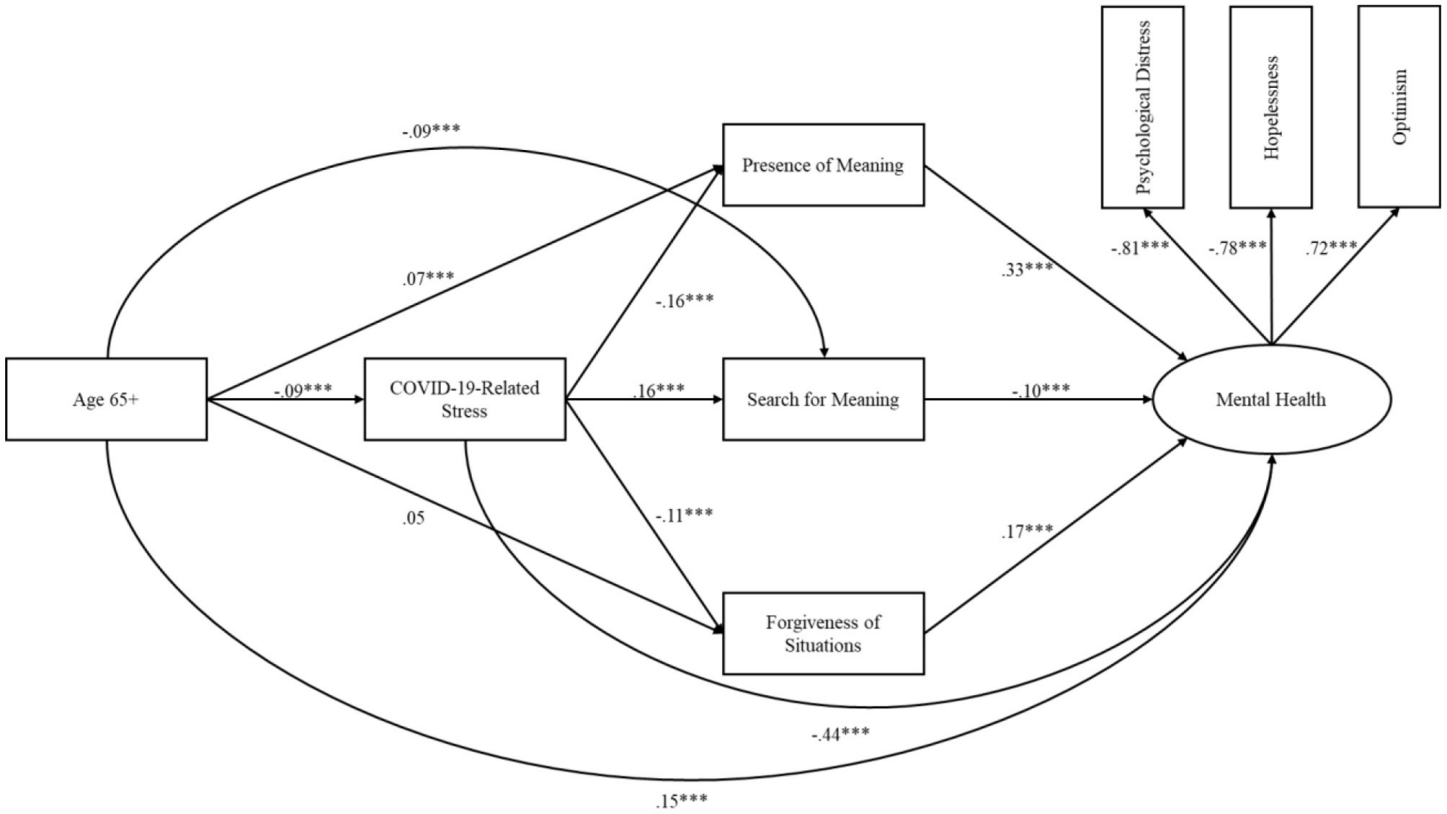
Results of structural equation model showing associations between older age, COVID-19-related stress perceptions, meaning, forgiveness, and mental health. Structural equation model of direct and indirect associations of older age with COVID-19-related stress perceptions, presence of and search for meaning, forgiveness of situations, and mental health. All coefficients are standardized and adjusted for sex, race, education, and income. ^***^p < 0.001.

The hypothesized serial mediation model was tested and results are summarized in Figure 2. To reduce measurement error and improve the precision of our structural estimates we created a latent mental health variable comprised of indicators including psychological distress, hopelessness, and optimism. Standardized loadings for psychological distress, hopelessness, and optimism were −0.81, −0.78, and 0.72, respectively. Overall model fit was good: χ^2^ = 52.39, *p* < 0.001; CFI = 0.99; RMSEA = 0.03; SRMR = 0.03. Participants age 65 and older showed less COVID-19-related stress perceptions (*B* = −0.16, β = −0.09, *p* < 001), more presence of meaning (*B* = 0.43, β = 0.07, *p* = 0.009), less search for meaning (*B* = −0.60, β = −0.09, *p* = 0.002), no relation to forgiveness of situations (*B* = 0.26, β = 0.05, *p* = 0.076), and better mental health (*B* = 1.15, β = 0.15, *p* < 0.001). COVID-19-related stress perceptions was related to less presence of meaning (*B* = −0.59, β = −0.16, *p* < 0.001), more search for meaning (*B* = 0.63, β = 0.16, *p* < 0.001), less forgiveness of situations (*B* = −0.35, β = −0.11, *p* < 0.001), and poorer mental health (*B* = −1.96, β = −0.44, *p* < 0.001). Presence of meaning (*B* = 0.39, β = 0.33, *p* < 0.001) and forgiveness of situations (*B* = 0.25, β = 0.17, *p* < 0.001) were related to better, and search for meaning (*B* = −0.11, β = −0.10, *p* < 0.001) was related to poorer mental health.

There are several indirect effects that are of interest in this study (see Table 3). There were seven single-mediator indirect associations to examine. Participants age 65 and older experienced less COVID-19-related stress perceptions which was associated with poorer mental health, and the indirect association was statistically significant (*B* = 0.32, β = 0.04, 95% CI of *B* = 0.15–0.50). Participants age 65 and older experienced less COVID-19-related stress perceptions which was associated with less presence of meaning, and the indirect association was statistically significant (*B* = −0.10, β = −0.01, 95% CI of *B* = −0.17 to −0.04). Participants age 65 and older experienced less COVID-19-related stress perceptions which was associated with more search for meaning, and the indirect association was statistically significant (*B* = 0.10, β = 0.01, 95% CI of *B* = 0.04–0.18). Participants age 65 and older experienced less COVID-19-related stress perceptions which was associated with less forgiveness of situations, and the indirect association was statistically significant (*B* = −0.06, β = −0.01, 95% CI of *B* = −0.11 to−0.02). Participants age 65 and older experienced more presence of meaning which was associated with better mental health, and the indirect association was statistically significant (*B* = 0.17, β = 0.02, 95% CI of *B* = 0.05–0.30). Participants age 65 and older experienced less search for meaning which was associated with poorer mental health, and indirect association was statistically significant (*B* = 0.07, β = 0.01, 95% CI of *B* = 0.02–0.13). Age was not associated with forgiveness of situations which was associated with better mental health, but the indirect association was not statistically significant (*B* = 0.06, β = 0.01, 95% CI of *B* = −0.01 to 0.14).

**Table 3:** Indirect associations.

**Indirect association**	**B**	**β**	**95% C.I**.	
			**LCL**	**UCL**
Age → COVID-19 stress perceptions → Mental health	0.32	0.04	0.15	0.50
Age → COVID-19 stress perceptions → Presence of meaning	−0.10	−0.01	−0.17	−0.04
Age → COVID-19 stress perceptions → Search for meaning	0.10	0.01	0.04	0.18
Age → COVID-19 stress perceptions → Forgiveness of situations	−0.06	−0.01	−0.11	−0.02
Age → Presence of meaning → Mental health	0.17	0.02	0.05	0.30
Age → Search for meaning → Mental health	0.07	0.01	0.02	0.13
Age → Forgiveness of situations → Mental health	0.06	0.01	−0.01	0.14
Age → COVID-19 stress perceptions → Presence of meaning → Mental health	0.04	0.00	0.02	0.07
Age → COVID-19 stress perceptions → Search for meaning → Mental health	0.01	0.00	0.00	0.02
Age → COVID-19 stress perceptions → Forgiveness of situations → Mental health	0.01	0.00	0.01	0.03

*LCL, UCL, lower and upper confidence limits*.

There were three serial (two-mediator) indirect associations to examine. Participants age 65 and older experienced less COVID-19-related stress perceptions which was associated with more presence of meaning which was associated with better mental health, and this serial indirect association was statistically significant (*B* = 0.04, β = 0.01, 95% CI of *B* = 0.02–0.07). Participants age 65 and older experienced less COVID-19-related stress perceptions which was associated with more search for meaning which was associated with poorer mental health, and this serial indirect association was statistically significant (*B* = 0.01, β = 0.01, 95% CI of *B* = 0.00–0.02). Participants age 65 and older experienced less COVID-19-related stress perceptions which was associated with more forgiveness of situations which was associated with better mental health, and this serial indirect association was statistically significant (*B* = 0.01, β = 0.01, 95% CI of *B* = 0.01–0.03).

## Discussion

The present study sought to examine direct and indirect pathways through which older age was associated with better mental health during the height of the first wave of the COVID-19 pandemic in the United States from April 2020 to July 2020. Our results provide the first known data linking age, COVID-19-related stress perceptions, meaning and forgiveness, and mental health. Older age was directly associated with less COVID-19-related stress perceptions, less search for meaning, and more presence of meaning and forgiveness of situations, and better mental health. Of equal interest in this study was the indirect association of older age with mental health through single and serial indirect pathways. Older age was associated with better mental health through single indirect pathways including COVID-19-related stress perceptions as well as presence of and search for meaning, but not forgiveness of situations. Older age was associated with better mental health through serial indirect pathways including older age and less COVID-19-related stress perceptions as the first association and COVID-19-related stress perceptions and each of the meaning and forgiveness variables as the second association in which each of these, in turn, was associated with mental health. In the case of both direct and indirect associations, the associations demonstrated in this model provide theoretical confirmation and empirical basis for continued examination in future studies.

More specifically, all of the hypothesized direct effects in this model were confirmed and were the most robust associations identified. Perhaps most importantly, the hypothesized direct associations between older age and lower COVID-19-related stress perceptions and better mental health were confirmed. This offers support for our theoretical model, grounded in lifespan theories (Erikson, 1982; Carstensen et al., 2003; Tornstam, 2005; Charles, 2010), suggesting that older individuals who develop stronger ego-integrity, altruistic perspectives, attention to positive vs. negative aspects of life, and social support may be able to appraise stressful situations in less threatening ways and enjoy better mental health. The present findings also coincide with existing empirical work on stress and mental health in general (Blazer and Hybels, 2005; Kessler et al., 2010a) and in specific reference to the COVID-19 pandemic (Carstensen et al., 2020; Klaiber et al., 2020; Kowal et al., 2020; Park et al., 2020; Knepple Carney et al., 2021; Varma et al., 2021).

Findings from this study also support empirical research regarding meaning and forgiveness. That is, the present findings confirm that, compared to younger individuals, older individuals experience higher presence of and lower search for meaning (Steger et al., 2009). The present findings also confirm the work of Kaleta and Mróz (2018) in Poland and show higher levels of forgiveness of situations in older U.S. adults. Existing empirical work has also shown that both meaning and forgiveness are connected with better mental health (Toussaint et al., 2015; Cohen et al., 2016; Musich et al., 2018), and the present findings add to this work, and in the case of forgiveness of situations, importantly contribute uniquely to the literature.

Testing the indirect associations of older age with mental health through COVID-19-related stress perceptions and meaning and forgiveness of situations revealed several unique findings. First, single-mediator models revealed that lower COVID-19-related stress perceptions and higher presence of and lower search for meaning all served as routes for indirect associations between older age and better mental health. The only hypothesized indirect effect that did not get supported was that older age was not connected to better mental health through higher levels of forgiveness of situations. Because this indirect association was being tested simultaneously with other indirect associations to mental health, this likely reflects the relative importance of meaning over forgiveness of situations in the context of the COVID-19 pandemic.

Examination of the serial indirect associations included in the model showed that all three hypothesized associations were confirmed. Older age was associated with lower COVID-19-related stress perceptions which was associated with more presence of and less search for meaning, and more forgiveness of situations, and presence of meaning and forgiveness of situations were associated with better mental health while search for meaning was associated with poorer mental health. Confirming the existence of these three indirect pathways from older age to better mental health is important. These findings offer what might be initial evidence of a model of positive aging that emphasizes the role of stress perceptions and positive psychological processes that promote mental health. This model could be expanded to include other positive psychological states and traits likely to develop in older age such as wisdom and awe (Krause and Hayward, 2015) to consider their role in the connection between older age and mental health. Other positive psychological variables such as grace, temperance, and curiosity could also be incorporated into this type of model.

## Limitations

Limitations regarding sample, measurement, and design and analysis of this study should be considered. First, despite the Harvard DLABSS panel being a diverse one (Strange et al., 2019), the respondents to this study were overwhelmingly White, highly educated, and middle-upper income. This is a considerable limitation, given that so much of the COVID-19 pandemic's impact was felt disproportionately by people of color and those with less education and income (McKnight-Eily et al., 2021). Nevertheless, data collection of this type without specific funding was only possible using tools such as the DLABSS. Second, our COVID-19-related stress perceptions variable was not a validated stress appraisal measure but was constructed by the authors to assess key areas of stress/concern in the face of the pandemic. While content- and face-valid, other reliability and validity statistics are not available. For a short measure, the internal consistency of the measure was acceptable, but further examination of this measure could be useful. At the time of our study we were unaware of any well-validated measures of this type for use. Third, regarding design and analysis, there are key considerations. The design is a cross-sectional one and causal conclusions cannot be made. The analyses are structural equation models that include several manifest and latent variables and multiple single- and serial-indirect associations. With all this competition for direct and indirect predictors of variance in the outcome, it is not surprising that several of the effect sizes (β) of the parameters are small. Nevertheless, effect sizes do not have to be large in size for them to have considerable impact on large populations or across a lifetime of years (Abelson, 1985; Prentice and Miller, 2003). It is also important to note that age 65+ variable is a dichotomous one in our model and while standardized parameter estimates are provided for interested readers, interpreting effect size using these estimates (β) should be done with caution (Gelman, 2008) and some analysts recommend placing equal or more emphasis on interpreting unstandardized coefficients in this case (Baguley, 2009; Hayes, 2017).

## Conclusions

The present study seeks to examine a model of older age, COVID-19-related stress perceptions, meaning and forgiveness, and mental health and better understand the multiple routes of connection that older age has to better mental health during the COVID-19 pandemic. The strongest routes are through reduced COVID-19-related stress perceptions and improved presence of meaning in life. However, more intricate connections do exist, and while smaller in magnitude, offer important avenues to consider in continued theoretical and empirical work. Furthermore, knowing that COVID-19-related stress perceptions and presence of meaning have been important mechanisms through which older adults might have experienced better mental health during the pandemic, and further, knowing that COVID-19-related stress perceptions and both meaning and forgiveness may act in direct, indirect and serial-indirect ways to promote mental health in older adults, it may be wise for gerontologists, counselors, and other practitioners to consider the implications of these findings, continue to observe theoretical and empirical developments in these areas, and envision how these findings may impact their own practice with older adults. As older adults continue to develop into one of the largest segments of our population and as the threat of more frequent pandemics in the future looms, all relevant tools for protecting vulnerable older adults from mental health impairments should come to the fore and be considered for possible use in the collection of tools for psychotherapeutic uses in these times. Of course, this will require further theoretical and empirical development, and we hope that the present study can serve as a beginning in that way.

## Data Availability Statement

The raw data supporting the conclusions of this article will be made available by the authors, without undue reservation upon reasonable request.

## Ethics Statement

The studies involving human participants were reviewed and approved by Human Research Protection Program, Harvard University. Written informed consent was not provided because informed consent was obtained electronically in the electronic survey.

## Author Contributions

DW and AC led data collection with all authors contributing to measure selection and survey development. AC cleaned, checked, and organized the database and codebook. LT performed the statistical analysis and wrote the first draft of the manuscript. All authors contributed to conception and design of the study, manuscript revision, read, and approved the submitted version.

## Conflict of Interest

The authors declare that the research was conducted in the absence of any commercial or financial relationships that could be construed as a potential conflict of interest.

## Publisher's Note

All claims expressed in this article are solely those of the authors and do not necessarily represent those of their affiliated organizations, or those of the publisher, the editors and the reviewers. Any product that may be evaluated in this article, or claim that may be made by its manufacturer, is not guaranteed or endorsed by the publisher.
